# A clinical-deep learning model for prolonged length of stay following endovascular aortic repair

**DOI:** 10.3389/fcvm.2026.1867707

**Published:** 2026-10-12

**Authors:** Changfu Hu, Tao Bai, Xiao Qin, Kangsui Tang, Tao Peng, Qingbo Yang, Minkang Jiang, Lin Zhang

**Affiliations:** 1 Department of Vascular Surgery, Nanxishan Hospital of Guangxi Zhuang Autonomous Region, Guilin, China; 2 Department of Vascular Surgery, The First Affiliated Hospital of Guangxi Medical University, Guilin, China

**Keywords:** abdominal aortic aneurysm, convolutional neural network, deep learning, endovascular aortic repair, predictive model

## Abstract

**Background:**

Prolonged length of stay (PLOS) after endovascular aortic repair (EVAR) for abdominal aortic aneurysm (AAA) is associated with increased complications, resource burden, and patient distress. Early identification of high-risk patients remains challenging. This study aimed to develop and externally validate a predictive model integrating clinical variables and three-dimensional deep learning (3D-DL) features from preoperative computed tomography angiography (CTA) to predict PLOS after EVAR.

**Methods:**

A total of 788 patients with AAA who underwent elective EVAR were enrolled from two hospitals. The internal training cohort consisted of 556 patients, and the external validation cohort included 232 patients. PLOS was defined as a LOS above the 75th percentile of the distribution. A clinical nomogram was developed using LASSO regression followed by multivariate logistic regression. Deep learning (DL) features were extracted from manually segmented computed tomography angiography (CTA) images using a 3D ResNet200 network, and LASSO regression was applied to select the most informative features. An ensemble model integrating the clinical predictors and the selected DL features was constructed. Model performance was assessed using receiver operating characteristic curves, calibration plots, and decision curve analysis.

**Results:**

LASSO combined with multivariate logistic regression identified five independent clinical predictors: age, hemoglobin, albumin, maximum aneurysm diameter, and diabetes. The clinical model achieved an area under the curve (AUC) of 0.917 in the internal cohort and 0.944 in the external cohort. From the 3D ResNet200 network, 32 DL features were extracted. LASSO regression further selected two of these features. The ensemble predictive model, which integrated the clinical predictors and DL features, demonstrated improved discrimination, with an AUC of 0.971 in the internal cohort and 0.958 in the external cohort. Internal validation with 1,000 bootstrap resamples yielded optimism-corrected AUCs of 0.911 for the clinical model and 0.966 for the ensemble model. Subgroup analyses demonstrated consistent model performance across age and diabetes strata, while performance in the female subgroup warrants further evaluation in larger cohorts.

**Conclusion:**

This study developed an ensemble predictive model to predict PLOS following EVAR. The model demonstrated excellent discrimination and provided significant prognostic value beyond clinical variables alone. This nomogram may serve as a preoperative tool for risk stratification and resource planning in this population. Future studies should focus on prospective validation and clinical integration.

## 1 Introduction

Abdominal aortic aneurysm (AAA), characterized by permanent dilation of the abdominal aorta. Its detection rate is increasing due to population aging and more widespread use of imaging screening (1). Endovascular aortic repair (EVAR) has become the preferred treatment for AAA patients who meet the criteria for surgical intervention. This minimally invasive approach is associated with shorter operative times, lower perioperative mortality, and reduced hospital stays compared to open surgical repair (2). Despite these advantages, substantial variability in postoperative recovery trajectories persists, and many patients continue to experience prolonged postoperative hospital stays, often termed prolonged length of stay (PLOS), which remains a significant clinical concern. PLOS not only increases the risk of in-hospital complications but also contributes to strained medical resources, increased financial burden for patients, and heightened doctor–patient conflicts (3).

PLOS is a widely used metric for assessing surgical outcomes and institutional efficiency, reflecting a complex interplay among disease severity, comorbidity profiles, psychosocial factors, and institutional characteristics (4). Previous studies on PLOS have largely focused on economic cost analyses or associations with particular postoperative complications (5, 6). In contrast, research aimed at early prediction of PLOS through the integration of disease-related features remains scarce, particularly in patients undergoing EVAR for AAA.

Recent advances in machine learning (ML) and deep learning (DL) have created new opportunities for extracting complex patterns from multidimensional data, thereby enabling automatic prediction with minimal human intervention. Conventional radiomics relies on handcrafted mathematical descriptors to quantify image intensity, shape, and texture. DL methods, especially three-dimensional (3D) convolutional neural networks, can instead learn hierarchical feature representations directly from volumetric medical images such as computed tomography angiography (CTA) (7). This capacity to derive high-level, abstract features without the need for predefined definitions is particularly promising for capturing subtle aneurysm characteristics that may influence postoperative recovery.

In this study, we developed and validated a DL-based model that integrates deep features extracted from pre-operative CTA with patient demographics, comorbidities, and operative variables to predict PLOS after EVAR. By leveraging multidimensional clinical and imaging information that is automatically learned from CTA data, we sought to provide a novel pre-operative tool for individualized risk assessment and hospital resource planning.

## 2 Materials and methods

### 2.1 Patients

This study is a retrospective clinical prediction analysis designed to identify predictive factors associated with PLOS in AAA patients undergoing EVAR.

Data were collected from patients diagnosed with AAA who underwent EVAR at the First Affiliated Hospital of Guangxi Medical University and Nanxishan Hospital of Guangxi Zhuang Autonomous Region between January 2014 and December 2025. Patient inclusion criteria were as follows: (a) age ≥18 years; (b) diagnosis of AAA confirmed by preoperative high-resolution CTA; (c) EVAR performed for an asymptomatic or non-ruptured AAA; and (d) availability of complete clinical data. Exclusion criteria included: (a) emergency EVAR for ruptured, symptomatic, traumatic, or mycotic AAA; (b) connective tissue disorders or infectious AAA; (c) coexisting malignant tumor or severe comorbidity with a life expectancy of less than one year; (d) missing clinical records or loss to follow-up; and (e) inadequate CTA image quality for deep learning analysis. A total of 906 patients who underwent EVAR at the two participating institutions were initially screened. After applying the inclusion and exclusion criteria, 118 patients were excluded, yielding a final cohort of 788 AAA patients. The internal training cohort comprised 556 patients from the First Affiliated Hospital of Guangxi Medical University, while the external validation cohort consisted of 232 patients from Nanxishan Hospital of the Guangxi Zhuang Autonomous Region.

This study was approved by the Ethics Committee of the First Affiliated Hospital of Guangxi Medical University (Approval No. 2023-E541-01) and the Ethics Committee of Nanxishan Hospital of Guangxi Zhuang Autonomous Region (Approval No. NXSYY-2026-057).

### 2.2 Data collection

The following demographic and clinical variables were collected: age, gender, body mass index (BMI), hemoglobin level (HGB), albumin level (ALB), smoking status, and history of hypertension, diabetes, coronary artery disease (CAD), peripheral artery disease (PAD), chronic obstructive pulmonary disease (COPD), chronic kidney disease (CKD), cerebrovascular disease (CVD), maximum aneurysm diameter, aneurysm neck length, aneurysm neck angle, aneurysm neck calcification, and length of stay (LOS). PLOS was defined as a LOS above the 75th percentile of the distribution (8).

In addition, preoperative CTA images of the AAA patients were collected. The images were acquired using multidetector row computed tomography systems from two manufacturers: either a Philips 64-row Brilliance iCT SP CT or a Canon 320-row Aquilion ONE ViSION CT. Scanning covered the region from the aortic arch to the femoral artery bifurcation. Key acquisition parameters included a tube voltage of 120 kV, tube current of 200 mA, detector collimation of 128  × 0.6 mm or 64  ×  0.625 mm, a field of view of 500 mm  × 500 mm, a rotation time of 330 ms, a matrix size of 512  × 512, a slice interval and thickness of 1 mm, and a reconstructed section thickness of 1 mm.

### 2.3 Construction of clinical predictive model

Clinical data from the internal training cohort and the external validation cohort were compared. A least absolute shrinkage and selection operator (LASSO) regression model was applied to identify predictors of PLOS and to construct a predictive nomogram (9, 10). Model performance was evaluated using receiver operating characteristic (ROC) curves and calibration curves. The area under the curve (AUC) was interpreted on a scale from 0.5 to 1.0. Internal validation was conducted via non-parametric bootstrap resampling (1,000 replicates) to compute the optimism-corrected area under the ROC curve (AUC). For each model, the apparent AUC, estimated optimism, and optimism-corrected AUC were reported. Calibration curves were generated to assess the agreement between predicted probabilities and observed outcomes. Decision curve analysis (DCA) was performed to determine the threshold for clinical net benefit of the prediction model.

### 2.4 Extraction of DL features

AAA segmentation was performed during the arterial phase of CTA imaging. Anonymized CTA images were analyzed using ITK-SNAP (version 3.8.0) (11). The region of interest (ROI) for AAA extended from 5 mm above the abdominal aorta dilation to the bifurcation of the common iliac artery (Figure 1). Manual segmentation was performed by a junior surgeon under the supervision of a senior surgeon, with any discrepancies resolved by the senior surgeon. Before inputting data into our network, we interpolated the original images to achieve uniform voxel spacing (1 mm × 1 mm × 1 mm). Using the Slope and Intercept values from the DICOM header, we converted CT images to Hounsfield Units (HU) and applied a cutoff to limit extreme values. Voxels below −200 HU and above 400 HU were clamped to the respective thresholds. We then calculated the mean and variance across all 3D images in the training cohort and normalized each image using Z-score normalization.

**Figure 1:**
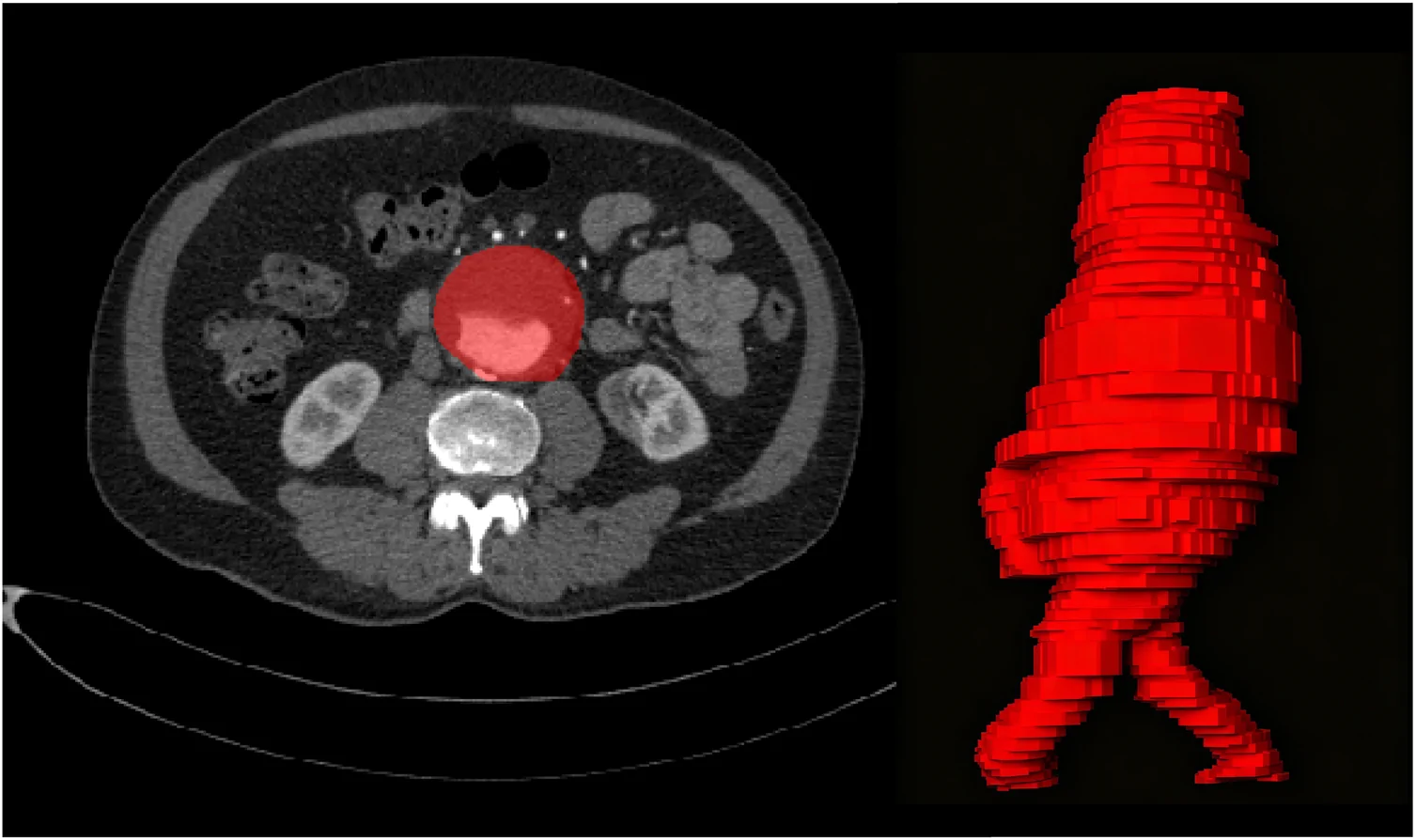
The regions of interest were precisely delineated on the anonymized CTA images using ITK-SNAP.

The DL model employed in this study utilized Residual Convolutional Neural Network (ResNet) 200 convolutional neural networks (CNN). The DL model was trained using 121 × 121 × 121 voxel bounding boxes from the ROI. A 3D fully convolutional network (FCN) composed of multiple 3D convolutional blocks was employed to classify the 3D inputs into binary outputs. The network was trained using stochastic gradient descent (SGD) with momentum (0.9), a cosine learning rate starting with an initial learning rate of 0.01, and a batch size of 4 over 96 epochs. The internal cohort was split into training (80%) and validation (20%) sets, with online data augmentation applied to the training set and early stopping triggered after 15 epochs without validation loss improvement. The model's performance was evaluated using the receiver operating characteristic (ROC) curve. After training, 32 DL features were extracted from the penultimate fully connected layer of the CNN.

### 2.5 Construction of ensemble predictive model

The extracted DL features were normalized through Z-score transformation to eliminate scale discrepancies (12). Subsequently, DL features with non-zero coefficients were retained using LASSO regression. The selected DL features were then integrated with the previously identified clinical predictors to construct an ensemble predictive model. The performance of this ensemble model was evaluated in both the internal training cohort and the external validation cohort using ROC curves, calibration curves, and DCA.

### 2.6 Statistical analysis

SPSS version 26.0, R version 4.2.1, and GraphPad Prism 9 were used for statistical analyses. Clinical data are presented as mean (SD) or median (P25, P75). Categorical variables were compared using the chi-square test or Fisher's exact test, while continuous variables were compared using Welch's two-sample t-test or the rank-sum test. A p-value < 0.05 was considered statistically significant.

Subgroup analyses were performed to evaluate the consistency of model performance across clinically relevant patient strata. For both the clinical and ensemble models, AUC values with 95% confidence intervals were computed separately for males and females, patients aged ≤75 years and >75 years, and patients with and without diabetes.

## 3 Results

### 3.1 Clinical characteristics

The clinical characteristics of the internal cohort and the external test cohort are summarized in Table 1. Significant differences were observed between the two cohorts in age and LOS (*p* < 0.001), while other variables were well balanced (*p* > 0.05). PLOS was defined as LOS >12 days, corresponding to the 75th percentile of the overall LOS distribution.

**Table 1:** Clinical characteristics of the internal and external test cohorts.

Characteristic	Cohort			*p*-value
	Overall *N* = 788	Internal Cohort *N* = 556	External Test Cohort *N* = 232	
LOS				<0.001
Mean ± SD	11.98 ± 5.40	11.28 ± 4.29	13.66 ± 7.17	
Median (Q1, Q3)	11.00 (10.00, 12.00)	11.00 (10.00, 12.00)	12.00 (11.50, 13.00)	
Age				<0.001
Mean ± SD	71 ± 6	72 ± 5	70 ± 7	
Median (Q1, Q3)	72 (67, 76)	72 (68, 76)	69 (64, 76)	
BMI				0.952
Mean ± SD	23.44 ± 2.78	23.36 ± 2.66	23.61 ± 3.03	
Median (Q1, Q3)	23.20 (21.60, 24.80)	23.20 (21.60, 24.80)	23.20 (21.45, 24.80)	
HGB				0.123
Mean ± SD	121 ± 11	122 ± 11	120 ± 11	
Median (Q1, Q3)	122 (114, 130)	122 (114, 131)	122 (113, 128)	
ALB				0.068
Mean ± SD	34.9 ± 3.1	34.7 ± 3.1	35.2 ± 3.0	
Median (Q1, Q3)	35.0 (33.0, 37.0)	35.0 (32.9, 37.0)	35.4 (33.3, 37.4)	
Aneurysm diameter				0.510
Mean ± SD	60 ± 9	60 ± 9	60 ± 10	
Median (Q1, Q3)	59 (53, 66)	59 (53, 66)	60 (54, 66)	
Neck length				0.135
Mean ± SD	28 ± 9	27 ± 9	28 ± 9	
Median (Q1, Q3)	27 (19, 36)	27 (19, 35)	29 (20, 37)	
Neck angulation				0.765
Mean ± SD	48 ± 16	48 ± 16	48 ± 17	
Median (Q1, Q3)	48 (34, 62)	48 (34, 62)	48 (34, 63)	
Gender				0.111
Male	701 (89.0%)	501 (90.1%)	200 (86.2%)	
Female	87 (11.0%)	55 (9.9%)	32 (13.8%)	
Smoking				0.307
No	269 (34.1%)	196 (35.3%)	73 (31.5%)	
Yes	519 (65.9%)	360 (64.7%)	159 (68.5%)	
Hypertension				0.416
No	292 (37.1%)	201 (36.2%)	91 (39.2%)	
Yes	496 (62.9%)	355 (63.8%)	141 (60.8%)	
Diabetes				0.439
No	646 (82.0%)	452 (81.3%)	194 (83.6%)	
Yes	142 (18.0%)	104 (18.7%)	38 (16.4%)	
CAD				0.539
No	599 (76.0%)	426 (76.6%)	173 (74.6%)	
Yes	189 (24.0%)	130 (23.4%)	59 (25.4%)	
PAD				0.516
No	583 (74.0%)	415 (74.6%)	168 (72.4%)	
Yes	205 (26.0%)	141 (25.4%)	64 (27.6%)	
COPD				0.055
No	740 (93.9%)	528 (95.0%)	212 (91.4%)	
Yes	48 (6.1%)	28 (5.0%)	20 (8.6%)	
CKD				0.668
No	669 (84.9%)	474 (85.3%)	195 (84.1%)	
Yes	119 (15.1%)	82 (14.7%)	37 (15.9%)	
CVD				0.406
No	493 (62.6%)	353 (63.5%)	140 (60.3%)	
Yes	295 (37.4%)	203 (36.5%)	92 (39.7%)	
Neck calcification				0.994
No	350 (44.4%)	247 (44.4%)	103 (44.4%)	
Yes	438 (55.6%)	309 (55.6%)	129 (55.6%)	

ALB, albumin; BMI, body mass index; CAD, coronary artery disease; CKD, chronic kidney disease; COPD, chronic obstructive pulmonary disease; CVD, cerebrovascular disease; HGB, hemoglobin; LOS, length of stay; PAD, peripheral artery disease.

Table 2 summarizes the clinical characteristics of the overall cohort stratified by PLOS status. Compared with the non-PLOS group, patients with PLOS were significantly older and had lower hemoglobin and albumin levels, a larger aortic diameter, and a higher prevalence of diabetes, hypertension, COPD, and female sex (*p* < 0.05). No significant differences were found for other variables (*p* > 0.05).

**Table 2:** Clinical characteristics of the PLOS and non-PLOS groups.

Characteristic	PLOS			*p*-value
	Overall *N* = 788	No *N* = 592	Yes *N* = 196	
Age				<0.001
Mean ± SD	71 ± 6	70 ± 6	75 ± 5	
Median (Q1, Q3)	72 (67, 76)	70 (65, 75)	75 (71, 80)	
BMI				0.283
Mean ± SD	23.44 ± 2.78	23.51 ± 2.85	23.21 ± 2.55	
Median (Q1, Q3)	23.20 (21.60, 24.80)	23.20 (21.60, 24.80)	23.00 (21.40, 24.75)	
HGB				<0.001
Mean ± SD	121 ± 11	124 ± 10	113 ± 11	
Median (Q1, Q3)	122 (114, 130)	125 (116, 132)	113 (105, 122)	
ALB				<0.001
Mean ± SD	34.9 ± 3.1	35.5 ± 2.9	33.0 ± 2.8	
Median (Q1, Q3)	35.0 (33.0, 37.0)	35.7 (33.8, 37.9)	33.7 (30.7, 35.3)	
Aneurysm diameter				<0.001
Mean ± SD	60 ± 9	58 ± 9	66 ± 8	
Median (Q1, Q3)	59 (53, 66)	57 (51, 64)	66 (60, 73)	
Neck length				0.294
Mean ± SD	28 ± 9	27 ± 9	28 ± 8	
Median (Q1, Q3)	27 (19, 36)	27 (19, 36)	29 (21, 35)	
Neck angulation				0.157
Mean ± SD	48 ± 16	48 ± 16	47 ± 16	
Median (Q1, Q3)	48 (34, 62)	49 (35, 62)	47 (33, 61)	
Gender				0.001
Male	701 (89.0%)	539 (91.0%)	162 (82.7%)	
Female	87 (11.0%)	53 (9.0%)	34 (17.3%)	
Smoking				0.230
No	269 (34.1%)	209 (35.3%)	60 (30.6%)	
Yes	519 (65.9%)	383 (64.7%)	136 (69.4%)	
Hypertension				0.001
No	292 (37.1%)	238 (40.2%)	54 (27.6%)	
Yes	496 (62.9%)	354 (59.8%)	142 (72.4%)	
Diabetes				<0.001
No	646 (82.0%)	512 (86.5%)	134 (68.4%)	
Yes	142 (18.0%)	80 (13.5%)	62 (31.6%)	
CAD				0.176
No	599 (76.0%)	443 (74.8%)	156 (79.6%)	
Yes	189 (24.0%)	149 (25.2%)	40 (20.4%)	
PAD				0.852
No	583 (74.0%)	437 (73.8%)	146 (74.5%)	
Yes	205 (26.0%)	155 (26.2%)	50 (25.5%)	
COPD				0.005
No	740 (93.9%)	564 (95.3%)	176 (89.8%)	
Yes	48 (6.1%)	28 (4.7%)	20 (10.2%)	
CKD				0.890
No	669 (84.9%)	502 (84.8%)	167 (85.2%)	
Yes	119 (15.1%)	90 (15.2%)	29 (14.8%)	
CVD				0.782
No	493 (62.6%)	372 (62.8%)	121 (61.7%)	
Yes	295 (37.4%)	220 (37.2%)	75 (38.3%)	
Neck Calcification				0.993
No	350 (44.4%)	263 (44.4%)	87 (44.4%)	
Yes	438 (55.6%)	329 (55.6%)	109 (55.6%)	

ALB, albumin; BMI, body mass index; CAD, coronary artery disease; CKD, chronic kidney disease; COPD, chronic obstructive pulmonary disease; CVD, cerebrovascular disease; HGB, hemoglobin; LOS, length of stay; PAD, peripheral arterial diseases.

### 3.2 Selection of clinical predictors and clinical predictive model performance

The candidate predictors were initially included in the model and subsequently reduced to five potential predictors using LASSO regression analysis, performed on the internal training cohort. The coefficients are presented in Supplementary Table S1. A cross-validated error plot of the LASSO regression model is shown in Figure 2A, and a coefficient profile is displayed in Figure 2B. The most regularized and parsimonious model—with a cross-validated error within one standard error of the minimum—included five variables: diabetes, age, albumin, hemoglobin, and maximum aneurysm diameter. As shown in Figure 2C, the ROC analysis of the abovementioned variables yielded AUC values greater than 0.5.

**Figure 2:**
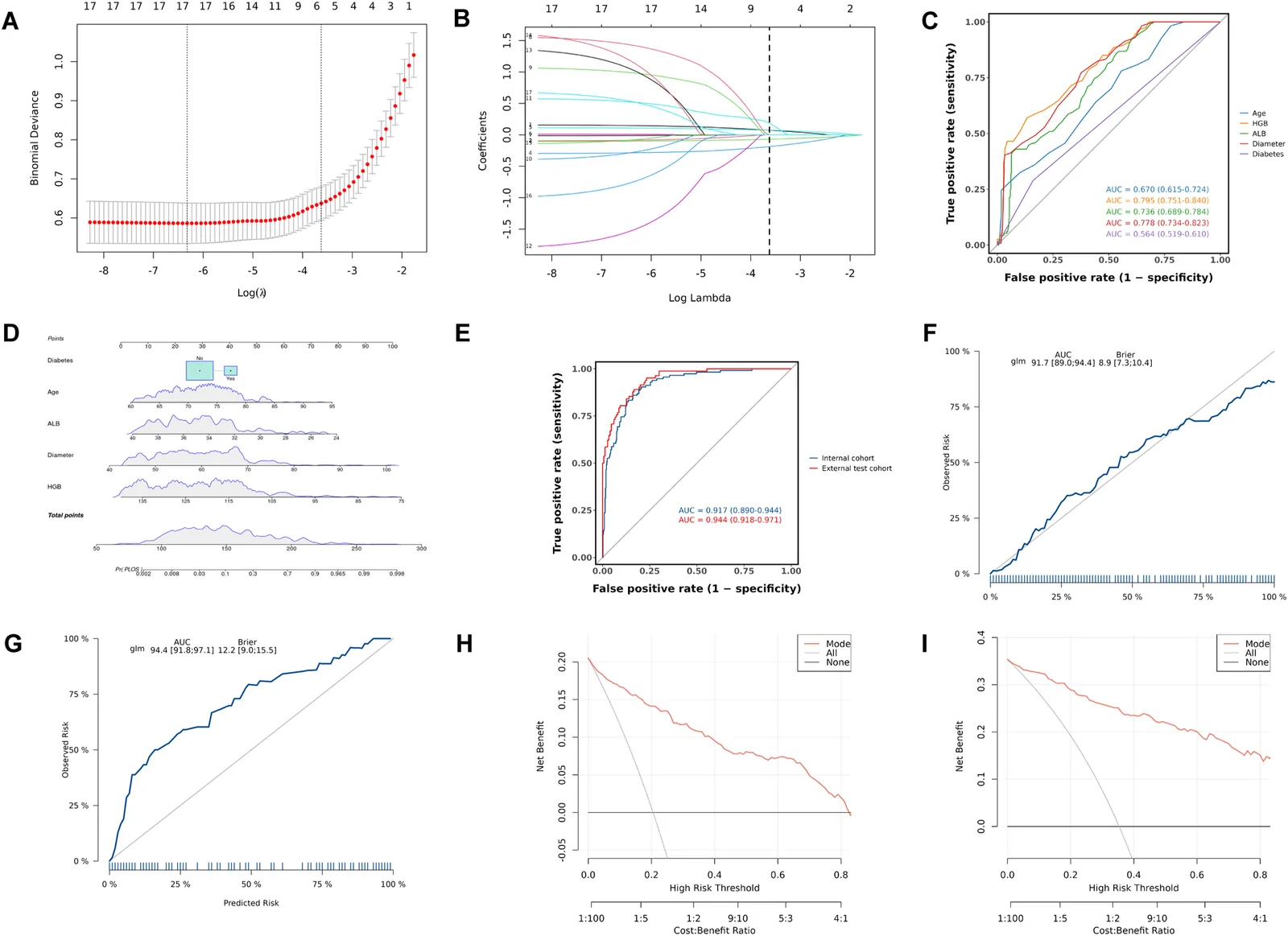
Construction and validation of the clinical predictive model. **(A)** Cross-validation plot of the LASSO regression model; **(B)** Coefficient path plot of the LASSO regression model. **(C)** ROC curve analysis of the candidate diagnostic indicators; **(D)** Nomogram for clinical predictive model; **(E)** ROC curves of the nomogram; **(F)** Calibration curve of the nomogram for the internal cohort. **(G)** Calibration curve of the nomogram for the external test cohort. **(H)** Decision curve analysis of the nomogram for the internal cohort. **(I)** Decision curve analysis of the nomogram for the external test cohort.

Further multivariate logistic analyses were carried out in training cohorts. Results are shown in Table 3. The final logistic model included 5 independent predictors (diabetes, age, albumin, hemoglobin, and maximum aneurysm diameter) and was developed as a simple-to-use nomogram, which is illustrated in Figure 2D. The performance of the model, as measured by the AUC, is presented in Figure 2E. In the internal cohort, the AUC was 0.917 (95% CI: 0.890–0.944). The model was further validated in an external cohort, achieving an AUC of 0.944 (95% CI: 0.918–0.971).

**Table 3:** Results of multivariate logistic regression for internal cohort in clinical predictive model.

Characteristic	*N*	Event N	OR	95% CI	*p*-value
Age	556	114	1.14	1.08, 1.22	<0.001
HGB	556	114	0.90	0.88, 0.93	<0.001
ALB	556	114	0.74	0.68, 0.81	<0.001
Diameter	556	114	1.11	1.08, 1.15	<0.001
Diabetes					
No	452	81	—	—	
Yes	104	33	2.06	1.08, 3.92	0.027

ALB, albumin; CI, Confidence Interval; HGB, hemoglobin; OR, Odds Ratio.

Internal validation using 1,000 bootstrap resamples yielded an apparent AUC of 0.917, an estimated optimism of 0.006, and an optimism-corrected AUC of 0.911.

The calibration plots for the nomogram in the internal and external test cohorts are presented in Figures 2F,G, respectively. Figures 2H,I show the decision curve analysis curves for the internal and external test cohorts based on the nomogram. Numerical calibration metrics, including Hosmer-Lemeshow test results, calibration intercept, and calibration slope, are reported in Supplementary Table S2.

### 3.3 DL feature selection and ensemble predictive model performance

Thirty-two DL features (DL0-DL31) were extracted from the fully connected layer of the CNN. Differences in these DL features between the internal cohort and the external test cohort are presented in Supplementary Table S3. The 32 DL features were then subjected to LASSO regression for feature selection. A cross-validated error plot of the LASSO regression model is shown in Figure 3A, with the corresponding coefficient profile displayed in Figure 3B. The coefficients are listed in Supplementary Table S4. Using the one-standard-error rule, the most regularized and parsimonious model retained two DL features: DL0 and DL1. These features were combined with clinical predictors to construct an ensemble predictive model. Figure 3C presents the ROC curves for the seven variables, each achieving an AUC greater than 0.5. Multivariate logistic regression was performed to assess whether these variables were independent predictors. The analysis revealed that DL0, DL1, diabetes, age, albumin, hemoglobin, and maximum aneurysm diameter were significantly associated with postoperative length of stay (PLOS) (*p* < 0.05, Table 4). These seven independent predictors were incorporated into the final logistic model, which was then used to construct a user-friendly nomogram (Figure 3D).

**Figure 3:**
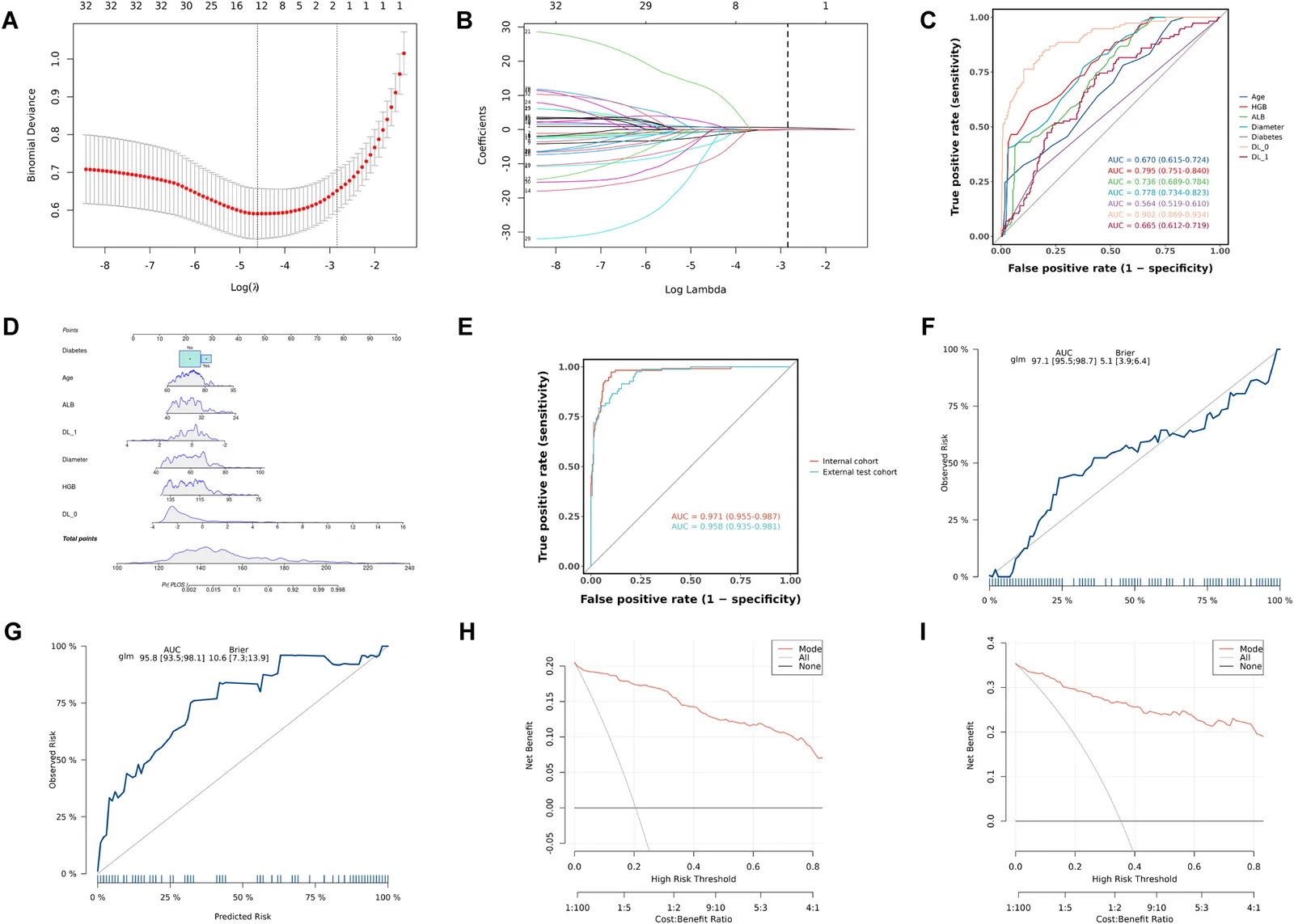
Construction and validation of the ensemble predictive model. **(A)** Cross-validation plot of the LASSO regression model; **(B)** Coefficient path plot of the LASSO regression model. **(C)** ROC curve analysis of the candidate diagnostic indicators; **(D)** Nomogram for ensemble predictive model; **(E)** ROC curves of the nomogram; **(F)** Calibration curve of the nomogram for the internal cohort. **(G)** Calibration curve of the nomogram for the external test cohort. **(H)** Decision curve analysis of the nomogram for the internal cohort. **(I)** Decision curve analysis of the nomogram for the external test cohort.

**Table 4:** Results of multivariate logistic regression for internal cohort in ensemble predictive model.

Characteristic	N	Event N	OR	95% CI	*p*-value
Age	556	114	1.13	1.05, 1.23	0.003
HGB	556	114	0.91	0.87, 0.94	<0.001
ALB	556	114	0.75	0.67, 0.85	<0.001
Diameter	556	114	1.12	1.07, 1.18	<0.001
Diabetes					
No	452	81	—	—	
Yes	104	33	2.95	1.17, 7.58	0.022
DL_0	556	114	2.29	1.87, 2.89	<0.001
DL_1	556	114	0.34	0.19, 0.58	<0.001

ALB, albumin; CI, Confidence Interval; DL, deeplearning; HGB, hemoglobin; OR, Odds Ratio.

The model achieved AUCs of 0.971 (95% CI: 0.955–0.987) in the internal cohort and 0.958 (95% CI: 0.935–0.981) in the external test cohort (Figure 3E). Internal validation using 1,000 bootstrap resamples yielded an apparent AUC of 0.971, an estimated optimism of 0.005, and an optimism-corrected AUC of 0.966. Calibration and decision curve analyses for both cohorts are presented in Figures 3F–I. Numerical calibration metrics are shown in Supplementary Table S2.

To formally assess the incremental predictive value of the DL features, we compared the clinical model and the ensemble model using the DeLong test for paired AUC comparison, continuous and categorical net reclassification improvement (NRI), and integrated discrimination improvement (IDI). In the internal validation cohort, the AUC improvement was statistically significant (*Δ*AUC = 0.054, DeLong *p* < 0.001), with a continuous NRI of 1.548 (*p* < 0.001) and an IDI of 0.337 (*p* < 0.001). In the external validation cohort, the AUC gain was more modest and borderline (*Δ*AUC = 0.014, DeLong *p* = 0.059); however, both the continuous NRI (1.246, *p* < 0.001) and IDI (0.219, *p* < 0.001) remained significant, indicating that the DL features improved risk reclassification even when the improvement in overall discrimination was limited.

The optimal probability threshold for the ensemble nomogram was determined using the Youden index on the internal training cohort, yielding a cut-off of 0.30. At this threshold, the ensemble model achieved a sensitivity of 91.2%, specificity of 93.7%, positive predictive value (PPV) of 78.8%, negative predictive value (NPV) of 97.6%, and overall accuracy of 93.2% in the internal cohort. In the external validation cohort, the corresponding values were 73.2%, 97.3%, 93.8%, 86.9%, and 88.8%, respectively. For the clinical nomogram, the Youden-optimal cut-off was 0.20, with performance metrics summarized in Supplementary Table S5.

### 3.4 Subgroup analysis

Subgroup analyses of the ensemble model are summarized in Supplementary Table S6. The ensemble model demonstrated consistent discriminative performance across age strata (≤75 years: AUC 0.952,95%CI 0.931–0.972; >75 years: AUC 0.960,95%CI 0.934–0.985) and diabetes status (no diabetes: AUC 0.961,95%CI 0.944–0.978; diabetes: AUC 0.941,95%CI 0.902–0.980). The clinical model showed a similar pattern of stability across these subgroups. In the sex-stratified analysis, the ensemble model achieved an AUC of 0.965 (95% CI 0.950–0.979) in males and 0.915 (95% CI 0.853–0.976) in females. When further stratified by center, the ensemble model performed well in females from the internal center (AUC 0.974) but showed reduced discrimination in females from the external center (AUC 0.859). However, the female subgroup sample size was limited, and the corresponding confidence intervals were wide, precluding definitive conclusions.

## 4 Discussion

### 4.1 Clinical value of the clinical predictive model for PLOS after EVAR

PLOS serves as both a surrogate marker for postoperative complications and inefficient resource utilization, as well as a patient-centered outcome reflecting the quality of perioperative care (13). In recent years, an increasing number of prediction models for PLOS have been developed across various surgical specialties. Ngui et al. demonstrated that integrating intraoperative parameters with preoperative variables significantly improved the ability to identify patients at risk of PLOS (14). Murase et al. developed a deep learning-based model that successfully predicted prolonged hospitalization after cardiovascular surgery using preoperative data (15). Similarly, in total hip and knee arthroplasty, LASSO-regression models have been developed and shown acceptable predictive performance (16, 17). These studies highlight the growing recognition that accurate preoperative identification of patients at risk of PLOS can facilitate risk stratification, guide resource allocation, and enable pre-emptive optimization of perioperative care.

Notably, however, no prediction model has yet been developed specifically to predict PLOS after EVAR in AAA patients. In contrast to open surgery, EVAR is associated with shorter hospital stays and faster recovery; nevertheless, inter-individual variability in postoperative recovery remains substantial, and PLOS continues to pose a significant clinical challenge. To address this gap, we first constructed a parsimonious clinical nomogram incorporating five easily obtainable preoperative variables—age, hemoglobin, albumin, maximum aneurysm diameter, and diabetes—to predict PLOS after elective EVAR. The model demonstrated good discrimination in both the internal training cohort (AUC = 0.917) and the external validation cohort (AUC = 0.944), with well-calibrated probability estimates and an optimism-corrected AUC of 0.911 after internal bootstrap validation.

The five predictors we identified have well-established relevance to EVAR outcomes in the existing literature. Advanced age is a well-established independent predictor of long-term survival after EVAR and a key factor influencing perioperative outcomes (18). Preoperative anemia reflects diminished oxygen-carrying capacity and limited physiological reserve. Anemic patients face an increased risk of postoperative myocardial ischemia and heart failure, which in turn extends hospitalization (19). The literature indicates that anemic patients have a significantly longer length of stay compared with non-anemic patients (9.2 vs. 6.3 days) and an approximately 2.7-fold higher odds of 30-day mortality after EVAR (18). Preoperative hypoalbuminemia, a surrogate for poor nutritional status, is significantly associated with prolonged hospitalization, increased complications, and higher mortality across a wide range of major surgical procedures (20). A larger maximum aneurysm diameter is not only a direct indicator of disease severity but also reflects more complex anatomy, often involving challenging neck morphology, greater thrombus burden, and increased inflammatory activity of the vessel wall (21). These factors complicate the endovascular procedure and prolong recovery. Studies have shown that a preoperative AAA diameter >59 mm is significantly associated with a higher risk of reintervention and mortality following EVAR (22). Studies have shown that patients with diabetes have worse early outcomes after EVAR. In a large retrospective study, diabetic patients experienced higher rates of postoperative myocardial infarction (1.0% vs. 0.6%) and 30-day readmissions (10.9% vs. 8.8%) compared with non-diabetic patients; insulin-dependent diabetic patients showed even more pronounced differences (1.9% vs. 0.7% for myocardial infarction (23). Furthermore, a comprehensive meta-analysis confirmed that diabetic patients undergoing EVAR have significantly higher 30-day mortality than their non-diabetic counterparts (24). Taken together, these lines of evidence are consistent with and support the findings of our study.

### 4.2 Incremental value of DL features from CTA

Deep learning has been increasingly applied to medical image analysis, enabling automated segmentation, feature extraction, and outcome prediction across various clinical contexts. For example, CNNs have demonstrated robust performance in segmenting brain tumors from multimodal MRI (25) and are widely used for organ and lesion segmentation in CT imaging (26, 27). A recent comprehensive review further highlighted the successful application of AI models in predicting a wide range of postoperative complications, including prolonged hospitalization in cardiovascular surgery populations (28).

In a previous study, we developed a prediction model for poor prognosis in AAA patients by integrating clinical data, 3D deep learning features, and immune cell data. Although that model demonstrated good performance, the data were derived from a single center and lacked external validation (29). Consequently, the application of 3D deep learning specifically to predict PLOS in patients undergoing EVAR for AAA remains extremely limited.

In this study, we trained a 3D ResNet200 model on manually segmented AAA regions to extract 32 DL features. These features were then refined using LASSO regression, yielding two robust predictors (DL0 and DL1). When combined with the five clinical variables, the resulting ensemble model demonstrated improved discrimination: the area under the curve (AUC) increased to 0.971 in the internal cohort and 0.958 in the external cohort (Figure 3E). The optimism-corrected AUC was 0.966, and calibration plots remained satisfactory (Figures 3F,G).

Subgroup analyses further supported the robustness of the ensemble model. The DL features provided consistent incremental discriminative value across age strata and diabetes status, suggesting that the image-derived features capture prognostic information that is not redundant with age- or diabetes-related clinical risk. This consistency across subgroups strengthens confidence that the DL features reflect genuine aneurysm characteristics rather than confounding by demographic or comorbidity variables.

The improvement in AUC and the low number of retained DL features are noteworthy. This suggests that the 3D CNNs effectively compressed the high-dimensional image information into a compact, highly predictive set of latent features. Unlike hand-crafted radiomic descriptors, which rely on predefined mathematical formulas, DL features are learned directly from the data and can encode complex patterns related to wall calcification, thrombus heterogeneity, periaortic fat texture, and subtle morphological variations that are difficult to quantify manually (30, 31).

The fact that only two DL features (DL0 and DL1)—selected from 32 extracted features by LASSO regression—were sufficient to significantly improve discrimination over the clinical model alone (DeLong *p* < 0.001, IDI = 0.337 in the internal cohort) suggests that preoperative CTA imaging contains compact yet highly informative prognostic signals that are not captured by conventional clinical variables. These results strongly support the concept that preoperative imaging harbors “hidden” prognostic information that complements traditional clinical risk factors.

The ensemble nomogram at the 30% cut-off demonstrated high specificity (93.7% internal; 97.3% external) and excellent NPV (97.6% internal; 86.9% external), suggesting that the model is particularly valuable for ruling out PLOS — patients predicted as low-risk by the nomogram have a very low probability of experiencing prolonged hospitalization. The moderate PPV (78.8% internal; 93.8% external) indicates that positive predictions should be interpreted in conjunction with clinical judgment, as false positives may occur. Clinicians may adjust the threshold based on local preferences: a lower cut-off (e.g., 10%) would maximize sensitivity for screening purposes, while a higher cut-off (e.g., 50%) would increase PPV for resource planning.

### 4.3 Calibration performance

Although the ensemble model exhibited excellent discrimination, we observed a consistent systematic underestimation of risk in the external validation cohort, as reflected by the calibration curve lying above the ideal line (Figure 3G). Numerical calibration assessment confirmed this pattern. The calibration intercept of the ensemble model in the external cohort was 1.633 (95% CI: 0.905 to 2.361, *p* < 0.001), indicating significant calibration-in-the-large with systematic risk underprediction. However, the calibration slope was 0.859 (95% CI: 0.634 to 1.085, *p* = 0.221), which was not significantly different from 1, suggesting that the model preserved the relative risk ordering across patients. The Hosmer–Lemeshow test also indicated significant miscalibration in the external cohort (*χ*^2^ = 120.07, df = 8, *p* < 0.001), although this test is known to be sensitive to sample size and should be interpreted with caution. In contrast, the model showed excellent calibration in the internal cohort (intercept = 0.005, slope = 1.007). Several factors may explain this calibration drift.

First, the training and validation cohorts differed significantly in age and baseline LOS (Table 1), with the external cohort being slightly younger but having a longer mean LOS. Higher LOS in the external cohort is likely attributable to differences in perioperative care pathways, discharge policies, or patient referral patterns between the two hospitals. Because PLOS was defined as a fixed threshold, the external cohort had a higher true event rate, causing the model to systematically underpredict risk. Second, certain unmeasured confounders that may influence length of stay—such as socioeconomic status, family support, and access to rehabilitation facilities—were not captured by our variables. Third, the relatively small external sample size may have introduced random variation in the calibration estimates.

We elected not to perform *post-hoc* intercept recalibration on the external validation cohort. This decision was guided by two considerations. First, external validation should serve as an honest assessment of model transportability without modifying model parameters using validation data. Second, recalibrating on a single external site would not guarantee transportability to a third institution and may obscure the clinically meaningful finding that hospital-level factors drive LOS variability—a finding that threshold redefinition alone cannot address. The choice of a single data-driven threshold (75th percentile) rather than multiple clinically defined cutoffs further reflects the absence of an established LOS benchmark for EVAR; sensitivity analyses across alternative thresholds would not resolve the fundamental challenge of institutional heterogeneity in discharge practices. Importantly, despite the observed calibration offset, decision curve analysis (Figures 3H,I) demonstrated that the ensemble model provides positive net benefit across a wide range of threshold probabilities, confirming its clinical utility for relative risk stratification.

In future work, we plan to prospectively collect data from additional collaborating centers, with a target of 50–100 consecutive EVAR cases per site and perform site-specific intercept recalibration — a straightforward procedure that adjusts only the baseline risk level without altering the relative contribution of each predictor. By calibrating the model across multiple institutions with diverse discharge practices and perioperative care protocols, we aim to quantify the magnitude and sources of calibration variability, establish standardized local calibration protocols, and ultimately derive a more robust, institution-adaptable prediction framework.

### 4.4 Comparison with existing risk stratification tools

Several general tools have been applied to patients undergoing AAA repair, including the American Society of Anesthesiologists (ASA) physical status classification, the National Surgical Quality Improvement Program (NSQIP) surgical risk calculator, and the Glasgow Aneurysm Score (GAS). Compared with these tools, the present model differs in its specific focus on PLOS after EVAR.

The ASA classification was developed in 1941 for general surgical risk stratification and has never been validated as a standalone predictor of PLOS after EVAR in any population (32). Its reported association with LOS in NSQIP-based studies (OR: 1.9, 95% CI: 1.6-2.3) is modest and reflects its role as a comorbidity summary rather than a dedicated prognostic tool for this specific outcome (33).

The NSQIP Surgical Risk Calculator is a comprehensive online tool that predicts 30-day outcomes, including length of stay, based on approximately 20 preoperative variables. However, the calculator was developed exclusively on a United States surgical population and is calibrated to US healthcare norms; direct application to our cohort was therefore not feasible. Moreover, an external validation study of the NSQIP calculator in aortic surgery reported suboptimal calibration and discrimination, concluding that its risk estimates were inaccurate for this patient population (34). Importantly, the NSQIP calculator's underlying model coefficients are not publicly available, precluding recalibration to non-US populations (35).

The GAS integrates age, shock, myocardial disease, cerebrovascular disease, and renal disease to predict mortality after AAA repair. It was not designed to predict length of stay. A previous study found no significant association between GAS and hospital stay duration after EVAR (*p* = 0.587) (36).

The present model, by incorporating both clinical variables and CTA-derived deep learning features, provides a tool specifically tailored to PLOS prediction in this population, although head-to-head comparisons with existing tools in prospective, multi-center cohorts are needed to confirm its relative utility.

### 4.5 Limitations

Several limitations should be acknowledged. The retrospective design may introduce selection bias, and unmeasured confounders such as frailty, socioeconomic status, and institutional discharge policies could affect LOS. Broader multi-center validation in diverse geographic and healthcare settings is warranted: the current external validation cohort came from a single hospital, and the number of PLOS events in the external cohort was relatively small, which may limit the precision of some performance estimates. The female subgroup was particularly small (*N* = 87; 11% of the total cohort), reflecting the known male predominance of AAA; although the ensemble model performed well in women from the internal center (AUC 0.974), reduced performance in the external center (AUC 0.859, *N* = 32) suggests that generalizability in female patients requires validation in larger, sex-balanced cohorts.

Additional limitations pertain to image analysis. AAA regions were manually segmented by a single surgeon under senior supervision; formal assessment of inter-observer and intra-observer segmentation variability was not performed. Although semi-automatic intensity-based tools and the high contrast of AAA on CTA may limit variability, the absence of a quantitative agreement metric remains a methodological limitation. Future studies should incorporate formal variability analyses and explore fully automated segmentation. Additionally, patient-level CT scanner metadata were not recorded, precluding formal cross-scanner comparisons; prospective studies should systematically document scanner identifiers to enable scanner-specific subgroup analyses.

The DL features (DL0 and DL1) were derived from a 3D ResNet200 feature extractor and lack direct anatomical interpretability. This is inherent to the feature-extraction design, which complicates standard Grad-CAM workflows. Ongoing work is pursuing 3D occlusion-based saliency mapping, SHAP-based feature attribution, and correlation with traditional radiomic features to establish convergent validity for the anatomical correlates of these representations.

Regarding statistical methodology, LASSO feature selection was performed on the entire training cohort, and standard bootstrap validation was applied only to the downstream logistic regression model, rather than employing a nested resampling framework. For the ensemble model, nested bootstrap was not feasible because the 32 DL features exhibited high pairwise collinearity, causing unstable LASSO feature subsets across bootstrap subsamples. Nevertheless, the independent external validation (AUC 0.958), in which the full pipeline was applied without modification to unseen data from a different institution and CT platform, provides a performance estimate unaffected by variable selection optimism. Future work should incorporate nested resampling for DL-augmented models using more efficient architectures.

## 5 Conclusions

This study developed an ensemble predictive model to predict PLOS following EVAR. The model demonstrated excellent discrimination and provided significant prognostic value beyond clinical variables alone. This nomogram may serve as a preoperative tool for risk stratification and resource planning in this population. Future studies should focus on prospective validation and clinical integration.

## Data Availability

The original contributions presented in the study are included in the article/Supplementary Material, further inquiries can be directed to the corresponding author/s.
