# Supplementary material for: A clinical-deep learning model for prolonged length of stay following endovascular aortic repair
Source: Front Cardiovasc Med. 2026 Oct 12;13:1867707. doi: 10.3389/fcvm.2026.1867707 (PMC13696232; doi:10.3389/fcvm.2026.1867707)
Supplement: Supplementary file 1 [file Datasheet1.docx]

Supplementary Table 1: LASSO regression coefficients for the candidate predictors

| variable | Coefficient |
| --- | --- |
| (Intercept) | 3.49092413 |
| Age | 0.07264935 |
| BMI | 0.00000000 |
| HGB | -0.06965921 |
| ALB | -0.18797349 |
| Diameter | 0.07410773 |
| Neck length | 0.00000000 |
| Neck angulation | 0.00000000 |
| Gender_Female | 0.00000000 |
| Smoking_Yes | 0.00000000 |
| Hypertension_Yes | 0.00000000 |
| Diabetes_Yes | 0.18481790 |
| CAD_Yes | 0.00000000 |
| PAD_Yes | 0.00000000 |
| COPD_Yes | 0.00000000 |
| CKD_Yes | 0.00000000 |
| CVD_Yes | 0.00000000 |
| Neck calcification _Yes | 0.00000000 |

ALB, albumin; BMI, body mass index; CAD, coronary artery disease; CKD, chronic kidney disease; COPD, chronic obstructive pulmonary disease; CVD, cerebrovascular disease; HGB, hemoglobin; LOS, length of stay; PAD, peripheral arterial diseases.

Supplementary Table 2: Numerical calibration metrics for the clinical and ensemble models in the internal and external validation cohorts.

| Model | Cohort | HLχ²  (df=8) | HL p | Calibration Intercept  (95% CI) | p | Calibration  Slope (95% CI) | p |
| --- | --- | --- | --- | --- | --- | --- | --- |
| Clinical | Internal | 22.01 | 0.005 | 0.518 (0.142  to 0.893) | 0.007 | 1.537 (1.254  to 1.820) | <0.001 |
| Clinical | External | 56.90 | <0.001 | 2.196 (1.399  to 2.994) | <0.001 | 1.716 (1.280  to 2.152) | 0.001 |
| Ensemble | Internal | 18.59 | 0.017 | 0.005 (−0.392  to 0.402) | 0.980 | 1.007 (0.808  to 1.207) | 0.943 |
| Ensemble | External | 120.07 | <0.001 | 1.633 (0.905  to 2.361) | <0.001 | 0.859 (0.634  to 1.085) | 0.221 |

HL, Hosmer–Lemeshow. A calibration intercept of 0 and slope of 1 indicates perfect calibration. Slope < 1 suggests overfitting; slope > 1 suggests underfitting.

Supplementary Table 3: DL features of the internal and external test cohorts

| **Characteristic** | **Cohort** | | | **p-value** |
| --- | --- | --- | --- | --- |
|  | **Overall  N = 788** | **Internal Cohort  N = 556** | **External Test Cohort  N = 232** |  |
| **DL_0** |  |  |  | 0.188 |
| Mean ± SD | -0.95 ± 2.32 | -0.99 ± 2.37 | -0.87 ± 2.20 |  |
| Median (Q1, Q3) | -1.75 (-2.39, -0.48) | -1.81 (-2.40, -0.61) | -1.63 (-2.34, -0.06) |  |
| **DL_1** |  |  |  | 0.919 |
| Mean ± SD | 0.13 ± 0.86 | 0.13 ± 0.86 | 0.13 ± 0.88 |  |
| Median (Q1, Q3) | 0.01 (-0.41, 0.64) | 0.01 (-0.41, 0.64) | 0.03 (-0.42, 0.60) |  |
| **DL_2** |  |  |  | 0.388 |
| Mean ± SD | 0.01 ± 0.42 | -0.01 ± 0.38 | 0.05 ± 0.50 |  |
| Median (Q1, Q3) | -0.08 (-0.19, 0.07) | -0.08 (-0.20, 0.06) | -0.08 (-0.17, 0.08) |  |
| **DL_3** |  |  |  | 0.013 |
| Mean ± SD | -0.01 ± 0.26 | -0.03 ± 0.26 | 0.02 ± 0.27 |  |
| Median (Q1, Q3) | -0.07 (-0.18, 0.10) | -0.09 (-0.19, 0.08) | -0.03 (-0.16, 0.14) |  |
| **DL_4** |  |  |  | 0.996 |
| Mean ± SD | 0.01 ± 0.16 | 0.01 ± 0.15 | 0.01 ± 0.16 |  |
| Median (Q1, Q3) | 0.02 (-0.08, 0.09) | 0.02 (-0.08, 0.09) | 0.02 (-0.08, 0.09) |  |
| **DL_5** |  |  |  | 0.966 |
| Mean ± SD | 0.01 ± 0.14 | 0.01 ± 0.13 | 0.00 ± 0.15 |  |
| Median (Q1, Q3) | 0.00 (-0.06, 0.07) | 0.00 (-0.06, 0.07) | 0.00 (-0.06, 0.06) |  |
| **DL_6** |  |  |  | 0.537 |
| Mean ± SD | 0.01 ± 0.08 | 0.00 ± 0.08 | 0.01 ± 0.08 |  |
| Median (Q1, Q3) | 0.01 (-0.01, 0.04) | 0.01 (-0.01, 0.03) | 0.01 (-0.01, 0.04) |  |
| **DL_7** |  |  |  | 0.800 |
| Mean ± SD | 0.00 ± 0.07 | 0.00 ± 0.07 | 0.00 ± 0.08 |  |
| Median (Q1, Q3) | 0.01 (-0.03, 0.04) | 0.01 (-0.03, 0.04) | 0.00 (-0.03, 0.04) |  |
| **DL_8** |  |  |  | 0.341 |
| Mean ± SD | 0.00 ± 0.05 | 0.00 ± 0.05 | 0.00 ± 0.05 |  |
| Median (Q1, Q3) | 0.00 (-0.03, 0.03) | 0.00 (-0.03, 0.03) | 0.00 (-0.03, 0.03) |  |
| **DL_9** |  |  |  | 0.938 |
| Mean ± SD | 0.00 ± 0.04 | 0.00 ± 0.03 | 0.00 ± 0.04 |  |
| Median (Q1, Q3) | 0.00 (-0.02, 0.01) | 0.00 (-0.02, 0.01) | 0.00 (-0.02, 0.01) |  |
| **DL_10** |  |  |  | 0.673 |
| Mean ± SD | 0.00 ± 0.04 | 0.00 ± 0.04 | 0.00 ± 0.04 |  |
| Median (Q1, Q3) | 0.00 (-0.01, 0.02) | 0.00 (-0.01, 0.02) | 0.00 (-0.01, 0.02) |  |
| **DL_11** |  |  |  | 0.569 |
| Mean ± SD | 0.000 ± 0.032 | 0.000 ± 0.031 | 0.000 ± 0.034 |  |
| Median (Q1, Q3) | 0.001 (-0.015, 0.019) | 0.001 (-0.015, 0.019) | 0.000 (-0.016, 0.018) |  |
| **DL_12** |  |  |  | 0.518 |
| Mean ± SD | 0.00 ± 0.03 | 0.00 ± 0.03 | 0.00 ± 0.03 |  |
| Median (Q1, Q3) | 0.00 (-0.02, 0.02) | 0.00 (-0.02, 0.02) | 0.00 (-0.02, 0.02) |  |
| **DL_13** |  |  |  | 0.243 |
| Mean ± SD | 0.001 ± 0.029 | 0.000 ± 0.028 | 0.002 ± 0.031 |  |
| Median (Q1, Q3) | 0.001 (-0.012, 0.012) | 0.000 (-0.012, 0.011) | 0.002 (-0.010, 0.012) |  |
| **DL_14** |  |  |  | 0.704 |
| Mean ± SD | 0.001 ± 0.031 | 0.000 ± 0.030 | 0.002 ± 0.032 |  |
| Median (Q1, Q3) | -0.003 (-0.016, 0.014) | -0.003 (-0.016, 0.014) | -0.003 (-0.016, 0.016) |  |
| **DL_15** |  |  |  | 0.686 |
| Mean ± SD | 0.001 ± 0.022 | 0.001 ± 0.021 | 0.000 ± 0.023 |  |
| Median (Q1, Q3) | 0.000 (-0.013, 0.013) | 0.000 (-0.013, 0.013) | -0.001 (-0.013, 0.013) |  |
| **DL_16** |  |  |  | 0.607 |
| Mean ± SD | 0.000 ± 0.021 | 0.000 ± 0.020 | -0.001 ± 0.022 |  |
| Median (Q1, Q3) | -0.001 (-0.012, 0.012) | -0.001 (-0.012, 0.012) | -0.001 (-0.013, 0.011) |  |
| **DL_17** |  |  |  | 0.573 |
| Mean ± SD | 0.001 ± 0.020 | 0.001 ± 0.019 | 0.001 ± 0.023 |  |
| Median (Q1, Q3) | 0.001 (-0.009, 0.011) | 0.002 (-0.009, 0.011) | 0.001 (-0.010, 0.010) |  |
| **DL_18** |  |  |  | 0.398 |
| Mean ± SD | 0.000 ± 0.020 | 0.001 ± 0.020 | -0.001 ± 0.021 |  |
| Median (Q1, Q3) | -0.001 (-0.012, 0.009) | -0.001 (-0.011, 0.010) | -0.002 (-0.012, 0.008) |  |
| **DL_19** |  |  |  | 0.564 |
| Mean ± SD | 0.000 ± 0.019 | 0.001 ± 0.018 | 0.000 ± 0.020 |  |
| Median (Q1, Q3) | 0.000 (-0.010, 0.012) | 0.000 (-0.010, 0.012) | -0.001 (-0.011, 0.012) |  |
| **DL_20** |  |  |  | 0.610 |
| Mean ± SD | -0.001 ± 0.017 | -0.001 ± 0.016 | 0.000 ± 0.018 |  |
| Median (Q1, Q3) | -0.001 (-0.008, 0.007) | -0.001 (-0.008, 0.006) | -0.001 (-0.008, 0.007) |  |
| **DL_21** |  |  |  | 0.173 |
| Mean ± SD | -0.001 ± 0.016 | -0.001 ± 0.016 | 0.000 ± 0.016 |  |
| Median (Q1, Q3) | -0.001 (-0.008, 0.008) | -0.001 (-0.009, 0.007) | 0.000 (-0.008, 0.009) |  |
| **DL_22** |  |  |  | 0.686 |
| Mean ± SD | 0.001 ± 0.014 | 0.001 ± 0.014 | 0.000 ± 0.015 |  |
| Median (Q1, Q3) | 0.001 (-0.008, 0.009) | 0.000 (-0.008, 0.009) | 0.002 (-0.008, 0.009) |  |
| **DL_23** |  |  |  | 0.848 |
| Mean ± SD | 0.000 ± 0.015 | 0.000 ± 0.014 | 0.000 ± 0.015 |  |
| Median (Q1, Q3) | 0.000 (-0.008, 0.007) | 0.000 (-0.008, 0.007) | 0.000 (-0.008, 0.007) |  |
| **DL_24** |  |  |  | 0.891 |
| Mean ± SD | 0.000 ± 0.014 | 0.000 ± 0.013 | 0.000 ± 0.015 |  |
| Median (Q1, Q3) | 0.000 (-0.007, 0.007) | 0.000 (-0.007, 0.007) | 0.001 (-0.008, 0.008) |  |
| **DL_25** |  |  |  | 0.227 |
| Mean ± SD | 0.001 ± 0.013 | 0.000 ± 0.013 | 0.002 ± 0.013 |  |
| Median (Q1, Q3) | 0.000 (-0.005, 0.007) | 0.000 (-0.005, 0.007) | 0.000 (-0.005, 0.009) |  |
| **DL_26** |  |  |  | 0.384 |
| Mean ± SD | 0.000 ± 0.012 | 0.000 ± 0.012 | -0.001 ± 0.013 |  |
| Median (Q1, Q3) | 0.000 (-0.007, 0.007) | 0.000 (-0.006, 0.007) | -0.001 (-0.008, 0.007) |  |
| **DL_27** |  |  |  | 0.730 |
| Mean ± SD | 0.000 ± 0.011 | 0.000 ± 0.011 | 0.000 ± 0.012 |  |
| Median (Q1, Q3) | 0.000 (-0.006, 0.006) | 0.000 (-0.006, 0.005) | 0.000 (-0.007, 0.006) |  |
| **DL_28** |  |  |  | 0.153 |
| Mean ± SD | 0.000 ± 0.011 | 0.001 ± 0.011 | 0.000 ± 0.011 |  |
| Median (Q1, Q3) | 0.000 (-0.006, 0.007) | 0.000 (-0.006, 0.007) | -0.001 (-0.006, 0.005) |  |
| **DL_29** |  |  |  | 0.397 |
| Mean ± SD | 0.000 ± 0.010 | 0.000 ± 0.010 | 0.001 ± 0.011 |  |
| Median (Q1, Q3) | 0.001 (-0.005, 0.006) | 0.000 (-0.006, 0.006) | 0.001 (-0.005, 0.006) |  |
| **DL_30** |  |  |  | 0.070 |
| Mean ± SD | 0.000 ± 0.009 | 0.000 ± 0.009 | -0.001 ± 0.010 |  |
| Median (Q1, Q3) | 0.001 (-0.006, 0.006) | 0.001 (-0.005, 0.006) | 0.000 (-0.007, 0.005) |  |
| **DL_31** |  |  |  | 0.444 |
| Mean ± SD | 0.000 ± 0.009 | 0.000 ± 0.009 | 0.000 ± 0.009 |  |
| Median (Q1, Q3) | 0.000 (-0.006, 0.005) | 0.000 (-0.006, 0.005) | 0.000 (-0.005, 0.006) |  |
|  | | | | |

Supplementary Table 4: LASSO regression coefficients for the DL features

| variable | Coefficient |
| --- | --- |
| (Intercept) | -1.03527544 |
| DL_0 | 0.53128849 |
| DL_1 | -0.04019347 |
| DL_2 | 0.00000000 |
| DL_3 | 0.00000000 |
| DL_4 | 0.00000000 |
| DL_5 | 0.00000000 |
| DL_6 | 0.00000000 |
| DL_7 | 0.00000000 |
| DL_8 | 0.00000000 |
| DL_9 | 0.00000000 |
| DL_10 | 0.00000000 |
| DL_11 | 0.00000000 |
| DL_12 | 0.00000000 |
| DL_13 | 0.00000000 |
| DL_14 | 0.00000000 |
| DL_15 | 0.00000000 |
| DL_16 | 0.00000000 |
| DL_17 | 0.00000000 |
| DL_18 | 0.00000000 |
| DL_19 | 0.00000000 |
| DL_20 | 0.00000000 |
| DL_21 | 0.00000000 |
| DL_22 | 0.00000000 |
| DL_23 | 0.00000000 |
| DL_24 | 0.00000000 |
| DL_25 | 0.00000000 |
| DL_26 | 0.00000000 |
| DL_27 | 0.00000000 |
| DL_28 | 0.00000000 |
| DL_29 | 0.00000000 |
| DL_30 | 0.00000000 |
| DL_31 | 0.00000000 |

Supplementary Table 5: Performance at Youden-optimal cut-offs for both models in both cohorts

| Model | Cohort | Cut-off | Sensitivity (%) | Specificity (%) | PPV (%) | NPV (%) | Accuracy (%) |
| --- | --- | --- | --- | --- | --- | --- | --- |
| Clinical | Internal | 20% | 86.0 | 82.4 | 55.7 | 95.8 | 83.1 |
| Clinical | External | 20% | 75.6 | 92.0 | 83.8 | 87.3 | 86.2 |
| Ensemble | Internal | 30% | 91.2 | 93.7 | 78.8 | 97.6 | 93.2 |
| Ensemble | External | 30% | 73.2 | 97.3 | 93.8 | 86.9 | 88.8 |

NPV: Negative Predictive Value；PPV: Positive Predictive Value

Supplementary Table 6: Subgroup analysis of model discrimination performance

| Subgroup | N (events) | Clinical AUC (95% CI) | Ensemble AUC (95% CI) | ΔAUC |
| --- | --- | --- | --- | --- |
| Male | 701 (162) | 0.915 (0.892–0.938) | 0.965 (0.950–0.979) | +0.050 |
| Female | 87 (34) | 0.907 (0.844–0.970) | 0.915 (0.853–0.976) | +0.007 |
| Age ≤75 | 581 (104) | 0.900 (0.870–0.930) | 0.952 (0.931–0.972) | +0.051 |
| Age >75 | 207 (92) | 0.908 (0.867–0.949) | 0.960 (0.934–0.985) | +0.052 |
| No diabetes | 646 (134) | 0.910 (0.884–0.937) | 0.961 (0.944–0.978) | +0.051 |
| Diabetes | 142 (62) | 0.903 (0.854–0.952) | 0.941 (0.902–0.980) | +0.038 |

AUC, area under the receiver operating characteristic curve; CI, confidence interval; ΔAUC, difference between the ensemble model and the clinical model AUC.
